# An Integrative Pharmacology-Based Pattern to Uncover the Pharmacological Mechanism of Ginsenoside H Dripping Pills in the Treatment of Depression

**DOI:** 10.3389/fphar.2020.590457

**Published:** 2021-02-15

**Authors:** Libin Zhao, Rui Guo, Ningning Cao, Yingxian Lin, Wenjing Yang, Shuai Pei, Xiaowei Ma, Yu Zhang, Yingpeng Li, Zhaohui Song, Wuxun Du, Xuefeng Xiao, Changxiao Liu

**Affiliations:** ^1^ School of Graduate, Tianjin University of Traditional Chinese Medicine, Tianjin, China; ^2^ Zhendong Research Institute, Shanxi Zhendong Pharmaceutical Co., Ltd, Beijing, China; ^3^ School of Life Sciences, Beijing University of Chinese Medicine, Beijing, China; ^4^ State Key Laboratory of Critical Technology in Innovative Chinese Medicine, TCM Research Center, Tianjin Tasly Pharmaceutical CO., LTD., Tianjin, China; ^5^ Shandong Huayu University of Technology, Shandong, China; ^6^ State Key Laboratory of Drug Delivery Technology and Pharmacokinetics, Tianjin Institute of Pharmaceutical Research, Tianjin, China

**Keywords:** ginsenoside H dripping pills, depression, network pharmacology, chronic unpredictable mild stress, cAMP signaling pathway

## Abstract

**Objectives:** To evaluate the pharmacodynamical effects and pharmacological mechanism of Ginsenoside H dripping pills (GH) in chronic unpredictable mild stress (CUMS) model rats.

**Methods:** First, the CUMS-induced rat model was established to assess the anti-depressant effects of GH (28, 56, and 112 mg/kg) by the changes of the behavioral indexes (sucrose preference, crossing score, rearing score) and biochemical indexes (serotonin, dopamine, norepinephrine) in Hippocampus. Then, the components of GH were identified by ultra-performance liquid chromatography-iron trap-time of flight-mass spectrometry (UPLC/IT-TOF MS). After network pharmacology analysis, the active ingredients of GH were further screened out based on OB and DL, and the PPI network of putative targets of active ingredients of GH and depression candidate targets was established based on STRING database. The PPI network was analyzed topologically to obtain key targets, so as to predict the potential pharmacological mechanism of GH acting on depression. Finally, some major target proteins involved in the predictive signaling pathway were validated experimentally.

**Results:** The establishment of CUMS depression model was successful and GH has antidepressant effects, and the middle dose of GH (56 mg/kg) showed the best inhibitory effects on rats with depressant-like behavior induced by CUMS. Twenty-eight chemical components of GH were identified by UPLC/IT-TOF MS. Subsequently, 20(*S*)-ginsenoside Rh2 was selected as active ingredient and the PPI network of the 43 putative targets of 20(*S*)-ginsenoside Rh2 containing in GH and the 230 depression candidate targets, was established based on STRING database, and 47 major targets were extracted. Further network pharmacological analysis indicated that the cAMP signaling pathway may be potential pharmacological mechanism regulated by GH acting on depression. Among the cAMP signaling pathway, the major target proteins, namely, cAMP, PKA, CREB, *p*-CREB, BDNF, were used to verify in the CUMS model rats. The results showed that GH could activate the cAMP-PKA-CREB-BDNF signaling pathway to exert antidepressant effects.

**Conclusions:** An integrative pharmacology-based pattern was used to uncover that GH could increase the contents of DA, NE and 5-HT, activate cAMP-PKA-CREB-BDNF signaling pathway exert antidepressant effects.

## Introduction

Depression is a mental disorder illness with a high disability, morbidity and recurrence rate (Chen et al., 2008). The main clinical features are decreased food-intake, low mood, anhedonia, activity decrease, irritability and other symptoms (Yang et al., 2018). In severe cases, they may have suicidal tendency (Zhu et al., 2020). An epidemiological multicenter study showed the odds of being depressed among cancer patients were more than five times higher than in the general population (Götze et al., 2020). Remarkably, comorbid depression in patients with cancer was compellingly established as a risk factor for suicide as well as rapid cancer progression (Shoval et al., 2019). There is no specific drug for the treatment of depression in cancer patients, and antidepressants are generally used. At present, the commonly used chemical antidepressants are mainly divided into four classes according to the chemical formula and the mechanism, including selective serotonin-re-uptake inhibitors (SSRI) such as sertraline and fluoxetine, serotonin and norepinephrine reuptake inhibitors (SNRI) like duloxetine and milnacipran, tricyclic anti-depressants (TCA) such as amitriptyline and imipramine, monoamine oxidase inhibitors (MAOI) like moclobemide and phenelzine (Zhao et al., 2015; Ostuzzi et al., 2018). There are several Chinese herbal formulae for the treatment of depression including Chaihu Shugan powder, Xiaoyao Pill and Shugan Jieyu capsule (Du et al., 2014; Fu et al., 2014; Wang et al., 2014). Clinically, although chemical antidepressants are widely used, their side effects are common, such as hepatotoxicity, drowsiness, sexual dysfunction, nausea, irritability and psychomotor impairment (Dai et al., 2010; Gorzalka and Hill, 2011; Zhu et al., 2020). Compared with chemical drugs, traditional Chinese medicine (TCM) are attracting more and more attention due to its advantages such as low toxicity and side effects, high safety, and high efficacy and toxicity reduction (Xie et al., 2018). Therefore, the research on the treatment of depression with TCM has certain clinical value.

Ginsenoside H dripping pills (GH), originated from Tasly Group, is the class 5 new traditional Chinese medicine which obtained from leaves of Panax Quinquefolium Linn by extracting, chemical degrading and chromatography separating (Chen et al., 2018). It is designed to be used for replenishing qi and blood, and is used as an adjuvant drug for treating cancer. The main ingredient of the drug is ginsenoside Rh2 and the content of ginsenoside Rh2 is about 30% (Ma et al., 2018). Early studies have indicated that ginsenoside Rh2 could significantly inhibit the growth of U14 cervical cancer bearing mouse (Zhang et al., 2013). Besides, ginsenoside Rh2 can significantly improve the depressive behavior of depressing mice (Wang et al., 2016). It can be said that GH not only has significant anti-tumor effects, but also has antidepressant effects, which provides exclusive drugs for the treatment of depression in cancer patients and has research value. However, it is still unclear that antidepressant effects and mechanism of GH. Therefore, the study adopted the integrated pattern of “pharmacodynamics - network pharmacological analysis - mechanism verification” to deeply study the antidepressant effects and potential mechanism of GH.

Chronic unpredictable mild stress (CUMS) model in rats was the closest animal model to clinical depression (Su et al., 2017). During the modeling process, rats which were subjected to different kinds of chronic unpredictable mild stress, fully simulated the social living environment of depressive patients and induced rats to produce many behavioral abnormal symptoms similar to those of depressive patients (Antoniuk et al., 2019; Li et al., 2019). For example, the reducing the sucrose preference in rats simulated the symptoms of anhedonia in depressive patients; the reducing the score of Open field exercise simulated the symptoms of low ability of autonomic movement in depressive patients (Tan et al., 2015; Liu et al., 2018). Therefore, the decrease of behavioral indexes (sucrose preference and open field exercise score) showed that CUMS depression model in rats were successfully established. In addition, the pathogenesis of depression is complex. Although several hypotheses about depression have been proposed, the monoamine hypothesis is still the most common hypothesis for depression, because most of the current antidepressants act on monoamine transporters or receptors (Auclair et al., 2013; Liu et al., 2020). The hypothesis believes that the occurrence of depression is mainly due to the lack of major monoamine neurotransmitters such as 5-hydroxytryptamine (5-HT), dopamine (DA) and norepinephrine (NE) in the central nervous system of the brain (Jesulola et al., 2018). Hence, the contents of 5-HT, DA and NE in hippocampus were decreased, which indicated that CUMS depression model in rats was successfully established. In conclusion, the antidepressant effects of GH were evaluated from the behavioral and biochemical indexes of depression model rats.

Network pharmacology was first proposed by Hopkins in 2007, which is based on disease-gene-target-drug interaction network to predict the material basis and mechanism of drug intervention in diseases (Li and Zhang, 2013). With the establishment of TCM database systems such as Encyclopedia of Traditional Chinese Medicine and Traditional Chinese Medicine Systems Pharmacology Database (Ru et al., 2014; Xu et al., 2019), network pharmacology has been widely used in TCM (Xu et al., 2014; Yu et al., 2017; Zhang et al., 2017; Li et al., 2018). Interestingly, the holistic philosophy of TCM is consistent with the key idea of emerging network pharmacology (Li et al., 2014). Our group had previously predicted the underlying pharmacological mechanism of Xueshuan-Xinmai-Ning (XXNT) acting on coronary heart disease (CHD) through network pharmacology method, and found that the XXNT in the treatment of CHD might be involved into the signal transduction in nervous-endocrine-immune-cardiovascular-metabolic system, and it is verified by experiments that XXNT plays a role in treating CHD via VEGF signal pathway (Mao et al., 2019). In addition, through network pharmacology analysis, it is found that Zhile alleviated depression-like behaviors by upregulating the cAMP-CREB-BDNF signaling pathway (Wu et al., 2019). Therefore, the pharmacological mechanism of the anti-depressant effects of GH could be evaluated by network pharmacology analysis.

**FIGURE 1:**
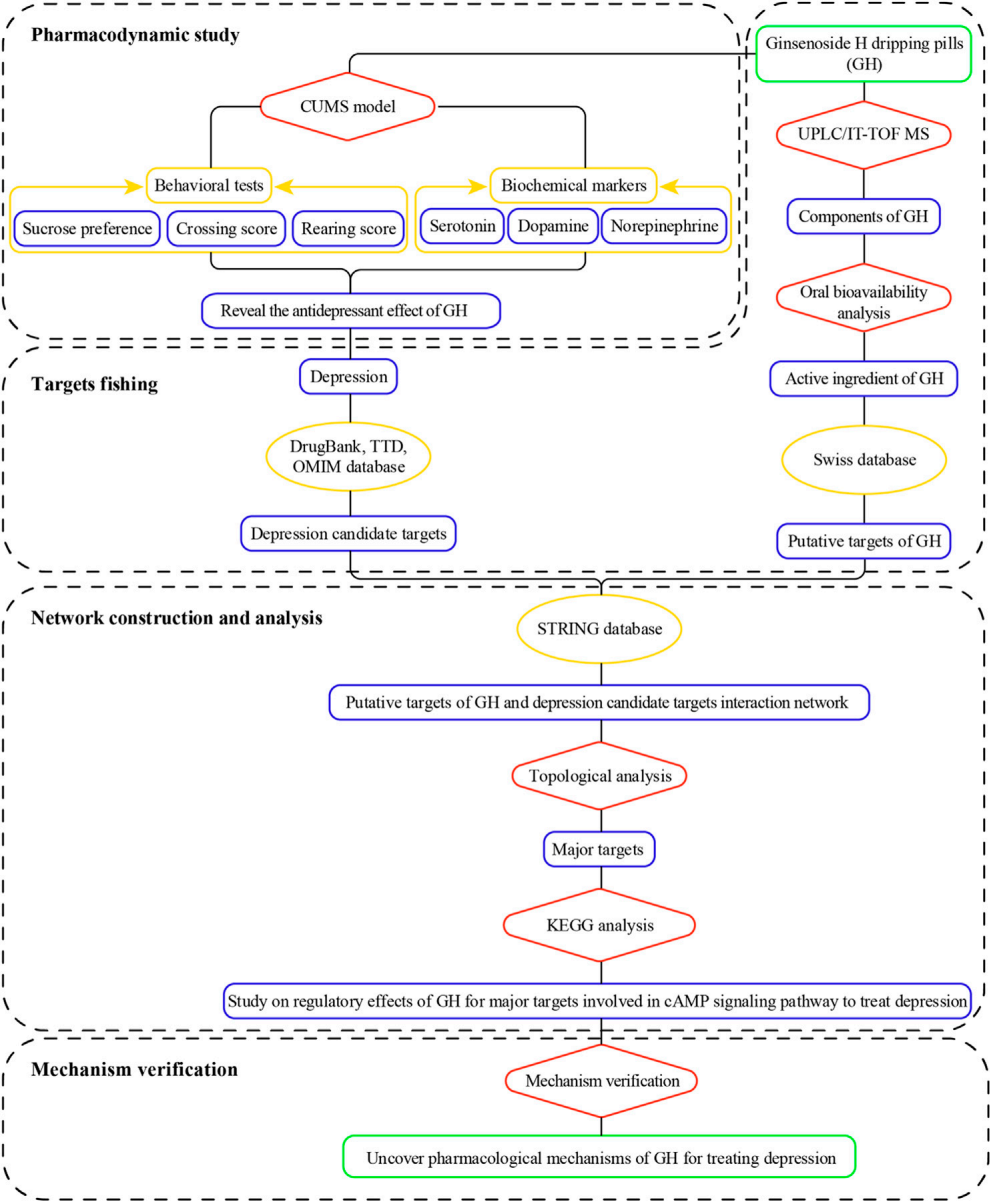
Flowchart of the study.

In this study, the integrated pharmacology-based pattern, which adopted pharmacodynamics-network pharmacology-mechanism verification, was used to elucidate the pharmacological mechanism of GH in treatment of depression. The antidepressant effects of GH were first confirmed by using chronic unpredictable mild stress model rats. The chemical components containing in GH were then identified by UPLC/IT-TOF MS, and the active ingredients of GH were further screened out based on OB and DL. Network pharmacology analysis was conducted to predict the potential pharmacological mechanism of GH in treatment of depression. At last, the results predicted by network pharmacology were further validated by western blotting and enzyme-linked immunosorbent assay. A flowchart of this study is illustrated in Figure 1.

## Materials and Methods

### Reagents and Materials

Bulk substance of Ginsenoside H dripping pills (GH) and GH (specification: 30 mg/pill, the total saponin content: 3.12 mg) were obtained from Tianjin Tasly Pharmaceutical Co., Ltd. (Batch NO. 20120606-16, 20160309, respectively, Tianjin, China). Fluoxetine hydrochloride (Flu) was provided by Suzhou Eli Lilly and Company (Suzhou, China). Chromatographic grade methanol and acetonitrile were obtained from Fisher Scientific Co. (Loughborough, United Kingdom). Pseudoginsenoside RT5, 20(*S*)-ginsenoside Rh1, 20(*R*)-ginsenoside Rh1, 20(*S*)-ginsenoside F1, ginsenoside Rh4, ginsenoside CK, 20(*S*)-ginsenoside Rh2, 20(*R*)-ginsenoside Rh2, isoginsenoside Rh3 standards were obtained from China National Institute for the Control of Pharmaceutical and Biological Products (Beijing, China). ginsenosides Rk2 standard was purchased from Chengdu Mansite Pharmaceutical Co., Ltd. (Chengdu, China). All standards were of at least 98% purity and were suitable for UPLC/IT-TOF analysis. The enzyme-linked immunosorbent assay (ELISA) kits, including serotonin (5-HT), dopamine (DA), norepinephrine (NE) and cyclic Adenosine monophosphate (cAMP) were supplied by Shanghai Lianshuo Biological Technology Co., Ltd. (Shanghai, China). The radioactive cyclic-AMP dependent protein kinase A (PKA) assay kit was purchased from Promega Corporation (Madison, Wisconsin, United States). The primary antibodies against brain derived neurotrophic factor (BDNF), cAMP-response element binding protein (CREB), phosphorylated cAMP-response element binding protein (*p*-CREB) and the secondary antibodies goat anti-rabbit IgG-HRP were purchased from Affinity Biosciences (Cincinnati, OH, United States). Bicinchoninic acid assay (BCA) kits was produced by Beyotime Institute of Biotechnology Co., Ltd. (Nanjing, China).

### Animals

A total of 80 Specific-pathogen free (SPF) male Sprague Dawley rats (180–220 g) were obtained from the China National Institutes for Food and Drug Control (SCXK (jing) 2017-0005). The rats were kept in an environmentally controlled room (temperature 22 ± 2°C, humidity 50 ± 10%, 12 h/12 h light/dark cycle) and were allowed to eat and drink freely. The laboratory animals were used according to requirements of the Ethics Committee of Tianjin University of Traditional Chinese Medicine (Tianjin, China), and the experimental methods were in line with the principles for protection of laboratory animals.

### Animal Grouping

After 7 days habituation, the rats were randomly divided into six groups (n = 10) according to the similar sucrose preference, crossing score and rearing score: the control group, CUMS group, low dose group of GH (28 mg/kg), middle dose group of GH (56 mg/kg), high dose group of GH (112 mg/kg), and Flu group (10 mg/kg).

### Establishment of Chronic Unpredictable Mild Stress (CUMS) Model and Drug Treatment

The CUMS procedure was carried out as described in the existing literatures (Zhong et al., 2018; Lu et al., 2019). The animals, except the control group, were separately placed and repeatedly exposed to a set of CUMS as follows: restraint stress (4 h), noise environment (110 dB, 1 h), electric shock to the foot (3 mA, one shock/5 s), tail clamp (tail nipped at 1 cm from the tip of the tail for 3 min), damp bedding (24 h), reversed light/dark cycle (24 h), high temperature stress (40°C, 20 min), ice-cold swimming (4°C, 5 min), 45° tilted cage (12 h), cage shaking (15 min), fasting food (24 h), water deprivation (24 h). Two stressors were applied every day and the whole stress procedure lasted for 5 weeks in a completely random order. During the modeling period, rats in the GH group and the Flu group were administrated with corresponding drugs; rats in the control group and the CUMS group were administrated with saline. Rats in all group were injected via gastric gavage at 10 ml/kg once daily.

### Behavioral Tests

#### Sucrose Preference Test

Sucrose preference test (SPT) was conducted at the day 0 and day 35 in accordance with previously described methods (Zhu et al., 2017). Briefly, 72 h before the test, the rats were bred individually two bottles 1% sucrose solution for 24 h, which were aimed to adapt to sucrose solution. Then, rats were exposed to one bottle of 1% sucrose solution and one bottle of water for 24 h. Finally, water and food were deprived for another 24 h. Sucrose preference test was conducted, in which rats were placed in separate cages and were freely access to two bottles containing sucrose solution (1%, w/v) and water, respectively. After 24 h, the weight of solution in every bottle was measured, and the rate of sucrose preference was calculated by the following formula:

$$Sucrose\;preference\;=\;\frac{sucrose\;consumtion\;}{water\;consumption+sucrose\;consumtion}\times 100\%$$

#### Open Field Test

The open field test (OFT) was carried out at day 0 and day 35 according to previously described methods (Dai et al., 2010). The activity of rats in each group were measured in a 100 cm × 100 cm × 50 cm box without ceiling, the inner wall and floor of which were coated with black paint. A video camera was used to record the rat behavior. The rats were released from the center of the arena, and were observed for 3 min. The following behavioral parameters were taken into the account: the crossing score (grid lines it crossed with at least three paws) and the rearing score (defined as standing upright with hind legs). To avoid the possible disturbance, the 75% alcohol was used to clean the floor box before each test.

### Hippocampus Sampling

The rats were sacrificed, 24 h after the behavioral tests. The whole brain was quickly dissected from the rats in ice-cold saline. The hippocampi were isolated on ice bath and immediately stored in liquid nitrogen for enzyme-linked immunosorbent assay and western blot analysis. All samples were stored at −80°C until assays.

### Preparation of Samples and Standard Solution

Bulk substance of GH was weighed 100 mg precisely. The powder was soaked in 20 ml methanol, extracted by ultrasonic at room temperature for 30 min, and precipitated to 25 ml volume. A stock solution containing ten standards (pseudoginsenoside RT5, 20(*S*)-ginsenoside Rh1, 20(*R*)-ginsenoside Rh1, 20(*S*)-ginsenoside F1, ginsenoside Rh4, ginsenoside CK, 20(*S*)-ginsenoside Rh2, 20(*R*)-ginsenoside Rh2, isoginsenoside Rh3 and ginsenoside Rk2) was prepared in methanol. All samples were filtered through 0.22 µm nylon membrane filters and the filtrate was analyzed directly by UPLC/IT-TOF.

### UPLC/IT-TOF Conditions

The UPLC analysis was performed on a Shimadzu LC-20A (Shimadzu, Kyoto, Japan) with a Waters Acquity UPLC HSS T3 column (2.1 × 100 mm, 1.8 μm). The column temperature was set at 35°C. The flow rate was set at 0.44 ml/min. The target sample temperature is set at 10°C and 5 μL of each sample was injected onto the column. The solvent system composed of mobile phase A (water) and mobile phase B (acetonitrile) in the following gradient: 0–9 min, 28–47%B; 9–17 min, 47–55%B; 17–22 min, 55–90%B; 22–24 min, 90–28%B; 24–25 min, 28–28%B. The experiment was performed on both ESI (+) ionization mode. The desolvation temperature was set at 200°C with desolvation gas flow set at 1.5 L/min. The capillary voltage was sat at 4.5 KV for ESI (+). The full scan data acquisition range was 100–1,300 Da. The LabSolutions-LCMS software (Shimadzu, Kyoto, Japan) were employed for data analysis and the chemical components of GH was identified.

### Targets Fishing

The chemical components containing in GH identified by UPLC/IT-TOF were filtered by integrating oral bioavailability (OB), drug similarity (DL) from Traditional Chinese Medicine Systems Pharmacology Database (TCMSP).^1^ The chemical components that meet both of the requirements OB ≥ 30%, DL ≥ 0.18, were retained as candidate active ingredients (Tao et al., 2013). The targets of candidate active ingredients were obtained from Swiss Target Prediction^2^ (Daina and Zoete, 2019). Species were selected as “Homo sapiens” and the targets with probability greater than 0 were predicted as the putative targets.

Known therapeutic targets acting on depression were collected from the DrugBank^3^ (Knox et al., 2011). Therapeutic Target Database (TTD)^4^ (Li et al., 2018) and Online Mendelian Inheritance in Man (OMIM) database^5^ (Hamosh et al., 2005) with the keyword “depression”. All targets enrolled in this research were human genes/proteins.

### Network Construction and Topological Analysis

Protein-protein interaction (PPI) data were obtained from STRING database^6^ (Szklarczyk et al., 2017) with setting species as “Homo sapiens” and the results were imported into the Cytoscape software (version 3.7.1, Boston, MA, United States) where the interaction network was constructed and analyzed. The topological features were calculated using Network Analyzer and the nodes with degree greater than twice the median degree of all nodes will be defined as major targets (Guo et al., 2020).

### Pathway Enrichment Analysis

To explore potential mechanism of predicted major targets, the Kyoto Encyclopedia of Genes and Genomes (KEGG) pathway enrichment analysis (Kanehisa et al., 2017) was performed using the Database Visualization and Integrated Discovery (DAVID)^7^ (Huang et al., 2007). KEGG pathways with enrichment *p* value less than 0.05 were employed for further study.

### Enzyme-Linked Immunoassay

Tissues from the rat hippocampus were homogenized by adding phosphate buffered saline (PBS) at pH 7.4 (Solarbio Science &Technology Co., Ltd., Beijing, China). Homogenate was centrifuged at 4°C for 10 min at 12,000 rpm to obtain the supernatant. The supernatant was separated and stored at −20°C until analysis. The concentration of 5-HT, DA, NE and cAMP were measured using commercially available ELISA kits in accordance with the manufacturer’s instructions. The absorbance at 450 nm was measured with a GloMax microplate reader (Promega, Madison, WI, United States), and the measured OD values were used to quantify the expression of the 5-HT, DA, NE and cAMP. All samples were determined three times repeatedly in the same assay to minimize inter-assay differences.

### Assay of PKA Activity

PKA activity was assayed using a radioactive PKA assay kit in accordance with the manufacturer’s instructions.

### Western Blot

Hippocampus tissue (approximately 50 mg) was solubilized by radio immunoprecipitation assay lysis buffer (Beijing, China) for 30 min on ice. The buffer contained 1% phenylmethylsulphonyl fluoride (Beyotime, China) and/or 1% phosphatase inhibitor. The pyrolysis products were clarified by centrifuging at 4°C for 15 min at 12,000 rpm. The supernatant was collected and the protein concentration was measured with the bicinchoninic acid protein assay kit. Proteins were separated by 10% sodium dodecyl sulfate polyacrylamide gel electrophoresis (SDS-PAGE) and then were transferred to polyvinylidene difluoride (PVDF) membranes, which were blocked in 5% nonfat milk or 5% BSA for 2 h and then washed three times with Tris Buffer Saline supplemented with 0.1% Tween-20 (TBST) buffer for 10 min each time. The membranes were incubated overnight at 4°C with primary antibodies BDNF (1:1,000 dilution), CREB (1:1,000 dilution), *p*-CREB (1:1,000 dilution), and rat polyclonal antibody GAPDH (1:1,000 dilution). Subsequently, the membranes were washed three times with TBST and were incubated for 2 h at room temperature with suitable goat anti-rabbit immunoglobulin G-horseradish peroxidase (IgG-HRP) secondary antibody (1:5,000 dilution). After rewashing with TBST, the immunoreactivity was observed using ECL reagent. The membranes were scanned by using an imaging system (Bio-Rad, Hercules, CA, United States) and the band strength was analyzed by using ImageJ software (National Institutes of Health, Bethesda, MD, United States).

### Statistical Analysis

Date were expressed using the mean ± the standard deviation (SD). SPSS version 21.0 software (IBM, Chicago, IL, United States) was used for statistical analysis. One-way analysis of variance (ANOVA) was used, and *p* value <0.05 was considered to be statistically significant.

## Results

### Establishment of CUMS Model and Antidepressant Effects of GH

In the study, CUMS rat model was established according to materials and methods. At the same time, the rats were intragastric administration. The antidepressant effects of GH were evaluated by the behavioral and biochemical indexes.

**FIGURE 2:**
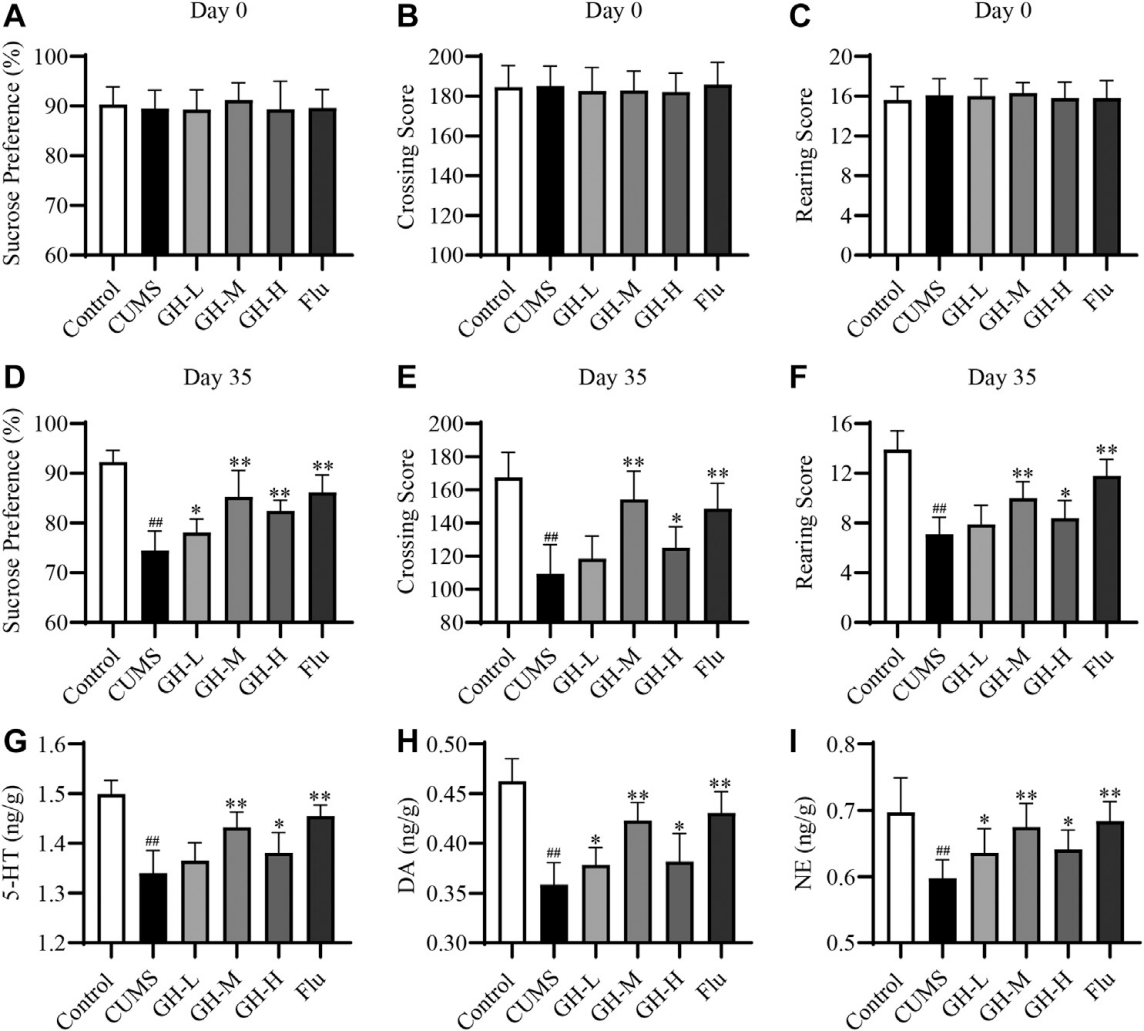
Effect of GH on CUMS rats in SPT, OET and neurotransmitters levels in hippocampus. **(A)** Sucrose preference of rats in each group at day 0. **(B)** Crossing score of rats in each group at day 0. **(C)** Rearing score of rats in each group at day 0. **(D)** Sucrose preference of rats in each group at day 35. **(E)** Crossing score of rats in each group at day 35. **(F)** Rearing score of rats in each group at day 35. **(G)** The concentration of 5-HT in each group. **(H)** The concentration of DA in each group. **(I)** The concentration of NE in each group. All data were expressed as mean ± standard error of mean (*n* = 10). ##*p* < 0.01 versus control group; *p < 0.05, *p < 0.01 versus CUMS group. GH-L (low dose group of GH), GH-M (middle dose group of GH), GH-H (high dose group of GH).

The results of behavioral indexes of rats in each group were shown in Figures 2A–F, the sucrose preference, the crossing score and the rearing score of rats in all groups were basically same at day 0 (*p* > 0.05, *p* > 0.05, *p* > 0.05, respectively). However, the sucrose preference, the crossing score and the rearing score of the CUMS group had a significant decrease compared with the control group at day 35 (*p* < 0.01, *p* < 0.01, *p* < 0.01, respectively). The changes of the sucrose preference, the crossing score and the rearing score of rats are typical characteristics of depression, which indicated that the establishment of depression model was successful. The sucrose preference of rats in low dose group of GH, middle dose group of GH, high dose group of GH, and Flu group were obviously higher than the CUMS group (*p* < 0.05, *p* < 0.01, *p* < 0.01, *p* < 0.01, respectively), showing the significant remission of depression symptoms. In the open field test, the crossing score and the rearing score of CUMS rat treated with middle dose of GH, high dose of GH, and Flu were markedly increased compared with the CUMS group (*p* < 0.01, *p* < 0.05, *p* < 0.01, respectively). However, there was no significant difference in crossing score and rearing score between low dose group of GH and the CUMS group (*p* > 0.05). Taken these results together, GH has antidepressant effects, and the middle dose of GH showed powerfully inhibitory effects on rats with depressant-like behavior induced by CUMS.

The results of biochemical indexes of rats in each group were demonstrated in Figures 2G–I. CUMS exposure significantly reduced the concentration of 5-HT, DA and NE (*p* < 0.01, *p* < 0.01, *p* < 0.01, respectively) compared with the control group, which indicated that the establishment of depression model was successful. The treatments with middle dose of GH, high dose of GH, and Flu significantly increased the concentration of 5-HT (*p* < 0.01, *p* < 0.05, *p* < 0.01, respectively), DA (*p* < 0.01, *p* < 0.05, *p* < 0.01, respectively) and NE (*p* < 0.01, *p* < 0.05, *p* < 0.01, respectively) compared with the CUMS group. Meanwhile, the treatments with low dose of GH significantly increased the DA (*p* < 0.05) and NE (*p* < 0.05) concentration compared to the CUMS group. However, low dose of GH had no significant effects on the 5-HT concentration in the CUMS-exposed rats (*p* > 0.05). Overall, the results indicated that middle dose of GH had better antidepressant effects. In the following mechanism verification experiment, the study mainly focused on the treatment group of GH at the middle dose (56 mg/kg).

### Identification of Chemical Components in GH by UPLC/IT-TOF MS

**FIGURE 3:**
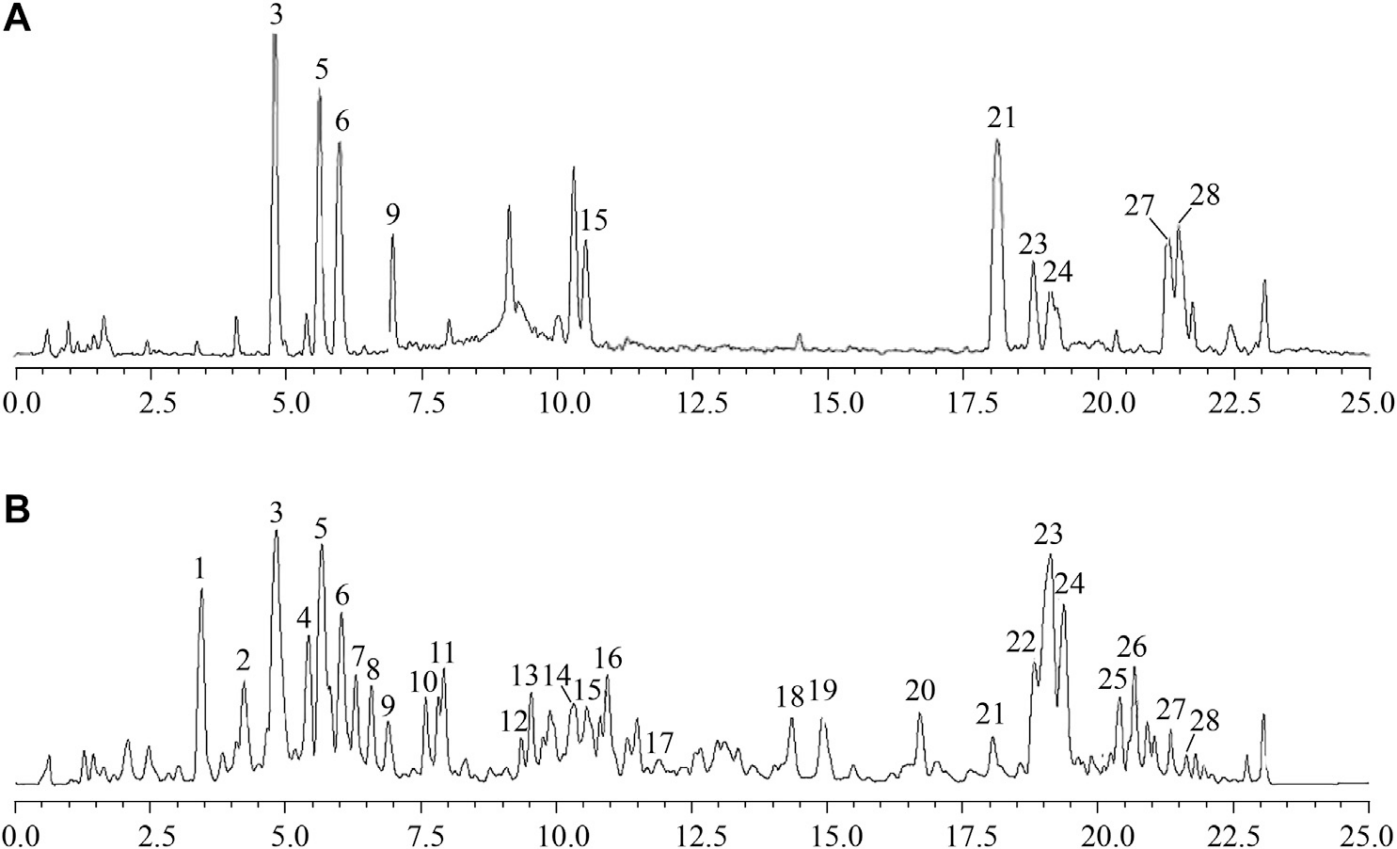
Total ion chromatogram (TIC) of GH sample **(A)** and mixed standard sample **(B)** in positive ion mode using UPLC/IT-TOF MS.

**FIGURE 4:**
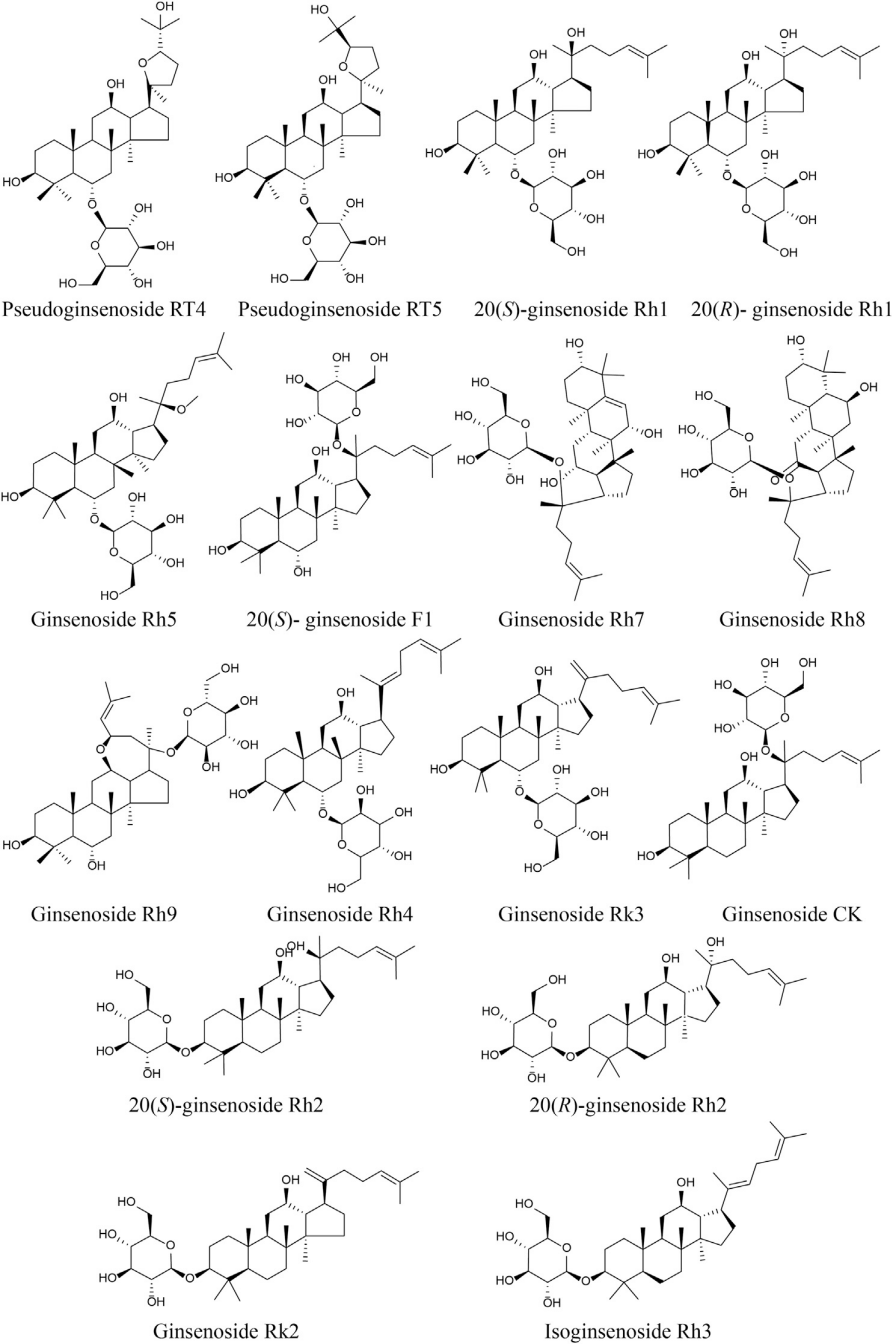
Structures of compounds in GH.

The UPLC/IT-TOF conditions was systemically optimized to receive good chromatographic separation and appropriate ionization. The total ion chromatogram (TIC) of the GH sample and mixed standard sample in the positive ion modes are, respectively, presented in Figures 3A,B. The 10 chemical components (component 3, 5, 6, 9, 15, 21, 23, 24, 27, 28) were identified as pseudoginsenoside RT5, 20(*S*)-ginsenoside Rh1, 20(*R*)-ginsenoside Rh1, 20(*S*)-ginsenoside F1, ginsenoside Rh4, ginsenoside CK, 20(*S*)-ginsenoside, 20(*R*)-ginsenoside Rh2, ginsenoside Rk2, isoginsenoside Rh3 by comparing the retention time, accurate and high-resolution mass and tandem mass spectra with chemical standards respectively. For the components without chemical standards, the molecular formula was established based on high precision quasi molecular ion such as [M+H]^+^, [2M+H]^+^or [M+Na]^+^ within a mass error of 5.0 ppm. Moreover, the MS^2^ information was used for confirming the structures of components by comparing the fragmentation regularity with ten standards or the related literatures (Patel et al., 2012; Zhu et al., 2018). Overall, a total of 28 chemical components were identified in GH and the related information of retention times and MS data was summarized in Table 1. The structures of 16 compounds related to 28 chemical components are displayed in Figure 4.

**TABLE 1:** Identification of chemical components in GH sample by UPLC/IT-TOF in positive ion mode.

No	tR (min)	Formula	Ion model	Theoretical mass	Measured mass	Error (ppm)	MS^2^	Components
1	3.429	C_30_H_54_O_6_	[M+H]^+^ [M+Na]^+^	511.3993,533.3813	511.3958,533.3778	−3.5–3.5	493.3876 [M+H-H_2_O]^+^ 475.3807 [M+H-2H_2_O]^+^ 457.3691 [M+H-3H_2_O]^+^ 439.3573 [M+H-4H_2_O]^+^ 421.3460 [M+H-5H_2_O]^+^	Unknown
2	4.206	C_36_H_62_O_10_	[M+H]^+^	655.4416	655.4391	−2.5	637.4292 [M+H-H_2_O]^+^ 619.4224 [M+H-2H_2_O]^+^ 475.3783 [M+H-H_2_O-glc]^+^ 457.3664 [M+H-2H_2_O-glc]^+^ 439.3567 [M+H-3H_2_O-glc]^+^ 421.3459 [M+H-4H_2_O-glc]^+^	Pseudoginsenoside RT_4_
3^a^	4.838	C_36_H_62_O_10_	[M+H]^+^ [M+Na]^+^	655.4416,677.4235	655.4390,677.4200	−2.6–3.5	457.3648 [M+H-2H_2_O-glc]^+^ 439.3565 [M+H-3H_2_O-glc]^+^ 421.3458 [M+H-4H_2_O-glc]^+^	Pseudoginsenoside RT_5_
4	5.402	C_36_H_62_O_9_	[M+H]^+^	639.4467	639.4463	−0.4	603.4249 [M+H-2H_2_O]^+^ 441.3694 [M+H-2H_2_O-glc]^+^ 423.3608 [M+H-3H_2_O-glc]^+^ 405.3521 [M+H-4H_2_O-glc]^+^	Isomer of ginsenoside Rh_1_
5^a^	5.655	C_36_H_62_O_9_	[M+Na]^+^	661.4286	661.4274	−1.2	621.4345 [M+H-H_2_O]^+^ 603.4256 [M+H-2H_2_O]^+^ 477.3926 [M+H-glc]^+^ 441.3687 [M+H-2H_2_O-glc]^+^ 423.3604 [M+H-3H_2_O-glc]^+^ 405.3518 [M+H-4H_2_O-glc]^+^	20(*S*)-ginsenoside Rh_1_
6^a^	5.799	C_36_H_62_O_9_	[M+Na]^+^	661.4286	661.4273	−1.3	621.4348 [M+H-H_2_O]^+^ 603.4248 [M+H-2H_2_O]^+^ 477.3911 [M+H-glc]^+^ 441.3699 [M+H-2H_2_O-glc]^+^ 423.3612 [M+H-3H_2_O-glc]^+^ 405.3519 [M+H-4H_2_O-glc]^+^	20(*R*)- ginsenoside Rh_1_
7	6.268	C_36_H_62_O_9_	[M+Na]^+^	661.4286	661.4275	−1.1	621.4347 [M+H-H_2_O]^+^ 603.4254 [M+H-2H_2_O]^+^ 441.3694 [M+H-2H_2_O-glc]^+^ 423.3606 [M+H-3H_2_O-glc]^+^ 405.3512 [M+H-4H_2_O-glc]^+^	Isomer of ginsenoside Rh_1_
8	6.566	C_36_H_60_O_9_	[M+H]^+^	637.4310	637.4283	−2.7	619.4206 [M+H-H_2_O]^+^ 457.3651 [M+H-H_2_O-glc]^+^ 439.3557 [M+H-2H_2_O-glc]^+^ 421.3463 [M+H-3H_2_O-glc]^+^ 403.3338 [M+H-4H_2_O-glc]^+^	Ginsenoside Rh_5_
9^a^	6.875	C_36_H_62_O_9_	[M+H]^+^	639.4467	639.4446	−2.1	621.4326 [M+H-H_2_O]^+^	20(*S*)- ginsenoside F_1_
							441.3684 [M+H-2H_2_O-glc]^+^	
							423.3609 [M+H-3H_2_O-glc]^+^	
10	7.562	C_36_H_60_O_9_	[M+H]^+^	637.4310	637.4297	−1.3	619.4240 [M+H-H_2_O]^+^ 457.3703 [M+H-H_2_O-glc]^+^ 439.3581 [M+H-2H_2_O-glc]^+^ 421.3461 [M+H-3H_2_O-glc]^+^	Ginsenoside Rh_7_/Rh_8_/Rh_9_
11	7.793	C_36_H_60_O_9_	[M+H]^+^	637.4310	637.4292	−1.8	619.4221 [M+H-H_2_O]^+^ 457.3666 [M+H-H_2_O-glc]^+^ 439.3565 [M+H-2H_2_O-glc]^+^ 421.3457 [M+H-3H_2_O-glc]^+^	Ginsenoside Rh_7_/Rh_8_/Rh_9_
12	9.327	C_36_H_60_O_8_	[M+H]^+^	621.4361	621.4349	−1.2	603.4246 [M+H-H_2_O]^+^ 441.3687 [M+H-H_2_O-glc]^+^ 423.3622 [M+H-2H_2_O-glc]^+^ 405.3535 [M+H-3H_2_O-glc]^+^	Ginsenoside Rh_4_/Rk_3_ or its isomer
13	9.517	C_36_H_60_O_8_	[M+Na]^+^	643.4180	643.4183	0.3	603.4253 [M+H-H_2_O]^+^ 441.3695 [M+H-H_2_O-glc]^+^ 423.3614 [M+H-2H_2_O-glc]^+^ 405.3521 [M+H-3H_2_O-glc]^+^	Ginsenoside Rh_4_/Rk_3_ or its isomer
14	10.321	C_36_H_60_O_8_	[M+H]^+^	621.4361	621.4380	1.9	441.3703 [M+H-H_2_O-glc]^+^ 423.3613 [M+H-2H_2_O-glc]^+^ 405.3521 [M+H-3H_2_O-glc]^+^	Ginsenoside Rh_4_/Rk_3_ or its isomer
15^a^	10.547	C_36_H_60_O_8_	[M+H]^+^	621.4361	621.4326	−3.5	603.4245 [M+H-H_2_O]^+^ 441.3698 [M+H-H_2_O-glc]^+^ 423.3612 [M+H-2H_2_O-glc]^+^ 405.3515 [M+H-3H_2_O-glc]^+^ 341.2817 [2glc+H_2_O-H]^+^	Ginsenoside Rh_4_
16	10.924	C_36_H_60_O_8_	[M+H]^+^	621.4361	621.4342	−1.9	603.4233 [M+H-H_2_O]^+^ 441.3699 [M+H-H_2_O-glc]^+^ 423.3612 [M+H-2H_2_O-glc]^+^ 405.3510 [M+H-3H_2_O-glc]^+^ 343.2977 [2glc+H_2_O+H]^+^ 325.2893 [2glc+H]^+^	Ginsenoside Rh_4_/Rk_3_ or its isomer
17	11.866	C_36_H_62_O_8_	[M+H]^+^	623.4517	623.4485	−3.2	605.4379 [M+H-H_2_O]^+^ 587.4325 [M+H-2H_2_O]^+^ 443.3880 [M+H-H_2_O-glc]^+^ 425.3761 [M+H-2H_2_O-glc]^+^ 407.3653 [M+H-3H_2_O-glc]^+^	Isomer of ginsenoside Rh_2_
18	14.333	C_36_H_62_O_8_	[M+H]^+^	623.4517	623.4484	−3.3	605.4428 [M+H-H_2_O]^+^ 587.4309 [M+H-2H_2_O]^+^ 425.3758 [M+H-2H_2_O-glc]^+^	Isomer of ginsenoside Rh_2_
19	14.912	C_36_H_60_O_8_	[M+H]^+^	621.4361	621.4331	−3.0	603.4266 [M+H-H_2_O]^+^ 585.4148 [M+H-2H_2_O]^+^ 441.3716 [M+H-H_2_O-glc]^+^ 423.3611 [M+H-2H_2_O-glc]^+^ 405.3511 [M+H-3H_2_O-glc]^+^	Ginsenoside Rh_4_/Rk_3_ or its isomer
20	16.699	C_36_H_60_O_8_	[M+H]^+^	621.4361	621.4345	−1.6	603.4254 [M+H-H_2_O]^+^ 585.4178 [M+H-2H_2_O]^+^ 441.3703 [M+H-H_2_O-glc]^+^ 423.3614 [M+H-2H_2_O-glc]^+^ 405.3509 [M+H-3H_2_O-glc]^+^	Ginsenoside Rh_4_/Rk_3_ or its isomer
21^a^	18.035	C_36_H_62_O_8_	[M+H]^+^ [M+Na]^+^	623.4517,645.4337	623.4482,645.4407	−3.5 7.0	605.4400 [M+H-H_2_O]^+^ 587.4305 [M+H-2H_2_O]^+^ 443.3866 [M+H-H_2_O-glc]^+^ 425.3755 [M+H-2H_2_O-glc]^+^ 407.3670 [M+H-3H_2_O-glc]^+^	Ginsenoside CK
22	18.809	C_36_H_62_O_8_	[2M+H]^+^	1,245.8962	1,245.8940	−2.2	587.4286 [M+H-2H_2_O]^+^ 425.3749 [M+H-2H_2_O-glc]^+^ 407.3656 [M+H-3H_2_O-glc]^+^	Isomer of ginsenoside Rh_2_
23^a^	19.127	C_36_H_62_O_8_	[M+Na]^+^ [2M+H]^+^	645.4337 1,245.8962	645.4323 1,245.8922	−1.4–4.0	605.4396 [M+H-H_2_O]^+^ 587.4279 [M+H-2H_2_O]^+^ 425.3741 [M+H-2H_2_O-glc]^+^ 407.3661 [M+H-3H_2_O-glc]^+^	20(*S*)-ginsenoside Rh_2_
24^a^	19.367	C_36_H_62_O_8_	[M+Na]^+^ [2M+H]^+^	645.4337 1,245.8962	645.4333 1,245.8895	−0.4–6.7	605.4403 [M+H-H_2_O]^+^ 587.4296 [M+H-2H_2_O]^+^ 425.3751 [M+H-2H_2_O-glc]^+^ 407.3652 [M+H-3H_2_O-glc]^+^	20(*R*)-ginsenoside Rh_2_
25	20.349	C_36_H_62_O_8_	[M+Na]^+^	645.4337	645.4316	−2.1	605.4405 [M+H-H_2_O]^+^ 587.4301 [M+H-2H_2_O]^+^ 443.3876 [M+H-H_2_O-glc]^+^ 425.3750 [M+H-2H_2_O-glc]^+^ 407.3667 [M+H-3H_2_O-glc]^+^	Isomer of ginsenoside Rh_2_
26	20.659	C_36_H_62_O_8_	[2M+H]^+^	1,245.8962	1,245.8914	−4.8	587.4309 [M+H-2H_2_O]^+^ 443.3858 [M+H-H_2_O-glc]^+^ 425.3751 [M+H-2H_2_O-glc]^+^ 407.3648 [M+H-3H_2_O-glc]^+^	Isomer of ginsenoside Rh_2_
27^a^	21.328	C_36_H_60_O_7_	[M+H]^+^	605.4412	605.4398	−1.4	587.4308 [M+H-H_2_O]^+^ 543.4216 [M+H-H_2_O-CO_2_]^+^ 443.3841 [M+H-glc]^+^ 425.3750 [M+H-H_2_O-glc]^+^ 407.3655 [M+H-2H_2_O-glc]^+^	Ginsenoside Rk_2_
28^a^	21.547	C_36_H_60_O_7_	[M+Na]^+^	605.4412	605.4426	1.4	587.4328 [M+H-H_2_O]^+^ 425.3748 [M+H-H_2_O-glc]^+^ 407.3657 [M+H-2H_2_O-glc]^+^	Isoginsenoside Rh_3_

a Accurately identified with reference standards.

### PPI Network Construction

Among the 16 compounds, only 20(*S*)-ginsenoside Rh2 satisfied the screening rules, OB ≥ 30% and DL ≥ 0.18. So, 20(*S*)-ginsenoside Rh2 was selected as active ingredient. Detailed information about OB and DL of 16 compounds was shown in Supplementary Table S1. The structure of 20(*S*)-ginsenoside Rh2 was used for predicting the putative targets in Swiss Target Prediction database. Totally, 43 putative targets of 20(*S*)-ginsenoside Rh2 containing in GH were predicted. Detailed target information about putative targets was shown in Supplementary Table S2. A total of 5 and 43 known therapeutic targets for depression were collected from DrugBank and Therapeutic Target Database (TTD) database, respectively, and 184 known targets of depression were obtained from OMIM database. In total, 230 depression candidate targets were enrolled after removing redundant entries. The detailed information is supplemented in Supplementary Table S3. The PPI network of the 43 putative targets of 20(*S*)-ginsenoside Rh2 containing in GH and the 230 depression candidate targets, was established based on STRING database, consisting of 184 nodes and 1,392 edges. Detailed information about this network was reflected in Supplementary Table S4.

### Network and Pathway Analysis

Network analyzer was employed to calculate the topological feature degree of the nodes in the PPI network. Nodes with degrees higher than two-fold median value of all nodes in the network (degree >23) were identified as the major targets. Consequently, 47 major targets were extracted. Among them, 29 targets were putative targets of 20(*S*)-ginsenoside Rh2 containing in GH, 32 targets were depression candidate targets, 15 targets were common targets, which were between putative targets of GH and depression candidate targets. The details were shown in Supplementary Table S5.

**FIGURE 5:**
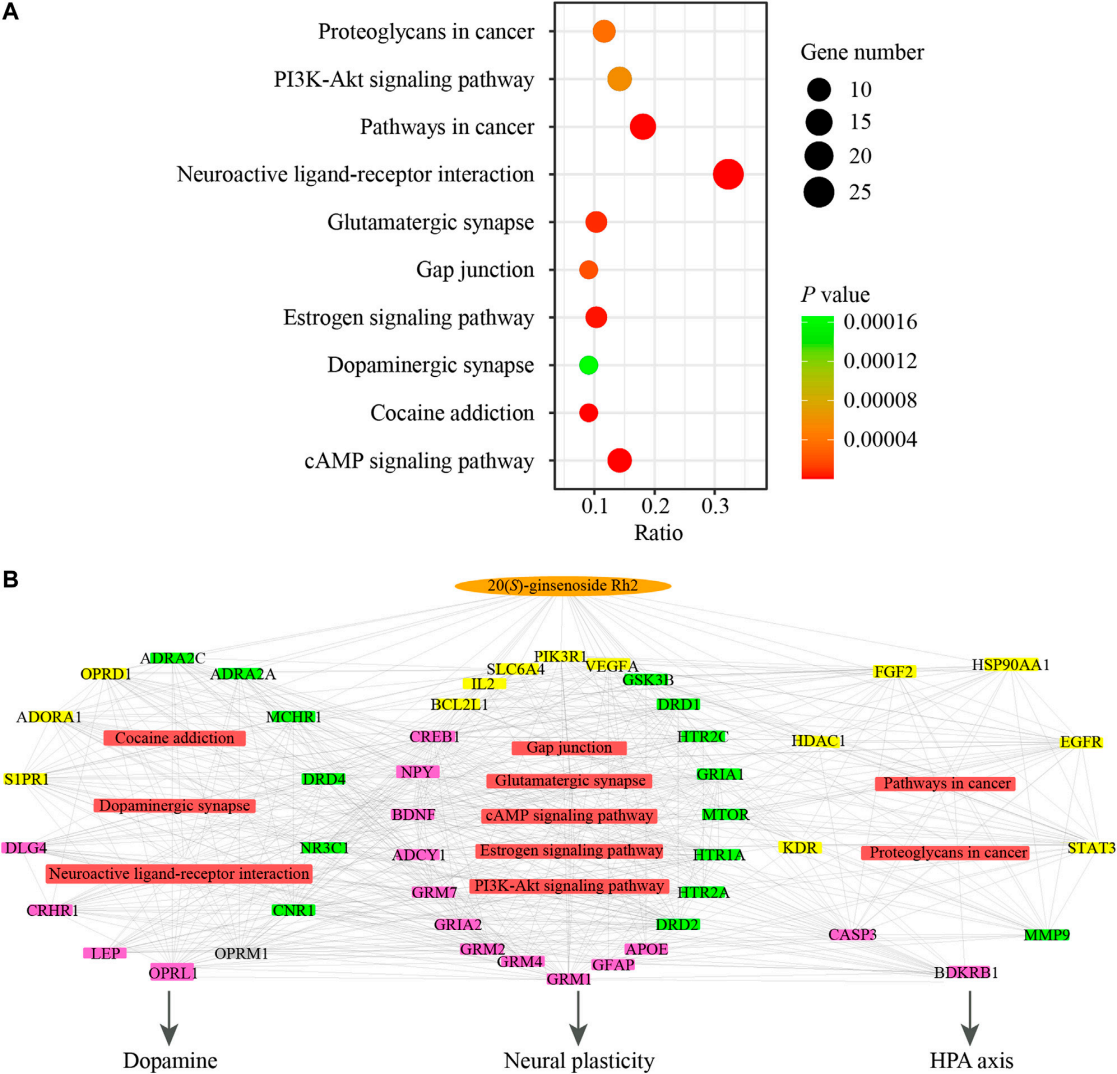
The network pharmacological results associating with GH in the treatment of depression. **(A)** KEGG pathway analysis of major targets. X-axis shows ratio and Y-axis shows involved pathways. Count and p value are shown on right. **(B)** Network of active ingredient in GH, major targets, the corresponding pathways and pharmagological effects. Orange oval nodes represent active ingredient in GH, yellow rectangle nodes represent related major targets of active ingredient in GH, pink rectangle nodes represent related major targets of depression, green rectangle nodes represent common major targets for both GH and depression, grey rectangle node represents other target, red rectangle nodes represent involved pathways from enrichment analysis of major targets, edges represent interactions among them.

In order to analyze the representative pathways related to the major targets, KEGG pathway analysis was performed to explore the potential pathways effected by GH and totally 45 significant pathways (*p* value <0.05) were obtained. The top 10 signal pathways were selected by *p* value for further study and were as shown in Figure 5A. The top 10 significant pathways could be divided into three functional modules, which were related to dopamine, hypothalamic-pituitary-adrenal (HPA) axis and neural plasticity respectively. Detailed information about results of pathways analysis was provided in Supplementary Table S6. Afterward a network consisted of the interactions between the active ingredient of GH, major targets, and top 10 significant pathways was constructed to illustrate the potential mechanism (Figure 5B). This network illustrated that GH may indirectly influence or directly interact with major targets which are involved in pathways related to HPA axis (such as pathways in cancer, proteoglycans in cancer), dopamine (such as neuroactive ligand-receptor interaction, cocaine addiction, dopaminergic synapse) and neural plasticity (such as cAMP signaling pathway, glutamatergic synapse, gap junction, PI3K-Akt signaling pathway and estrogen signaling pathway) to achieve the antidepressant effects.

### Experimental Validation of Major Targets and Pathway

**FIGURE 6:**
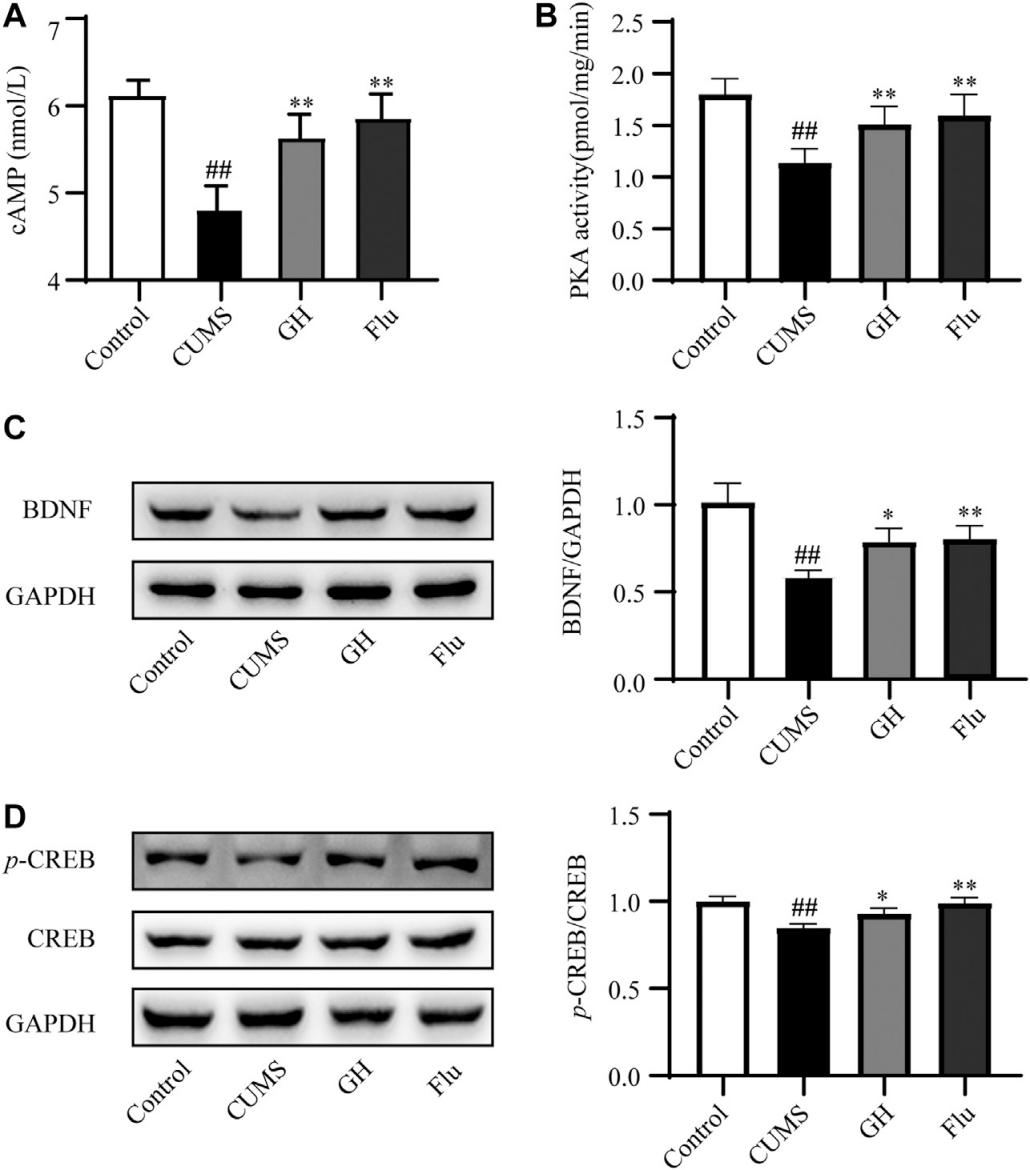
Experimental validation of target proteins involed in cAMP signaling pathway in the hippocampus of CUMS-induced rats. **(A)** Effect of GH treatment on cAMP concentrations (*n* = 10). **(B)** Effect of GH treatment on PKA activity (*n* = 10). **(C)** Effect of GH treatment on BDNF expression. **(D)** Effect of GH treatment on p-CREB/CREB ratio. All data were expressed as mean ± standard error of mean. ##*p* < 0.01 versus control group; *p < 0.05, **p < 0.01 versus CUMS group.

To explore the effects of GH on the cAMP pathway, we examined the concentration of cAMP via ELISA, PKA activity using a radioactive PKA assay kit, and protein expression levels of BDNF, CREB, *p*-CREB in the hippocampus of each group rats by Western blot (Figure 6). Compared with the control group, a significant decrease in cAMP concentration and PKA activity was observed in the hippocampus of the CUMS group (*p* < 0.01, *p* < 0.01, respectively), but daily administration of GH (56 mg/kg) or fluoxetine (10 mg/kg) obviously increased cAMP concentration and PKA activity in the hippocampus (*p* < 0.01, *p* < 0.01, respectively) compared with the CUMS rats. Meanwhile, BDNF expression levels and the *p*-CREB/CREB ratio in the hippocampus of the CUMS group were decreased (*p* < 0.01, *p* < 0.01, respectively). Following treatment with GH (56 mg/kg) or fluoxetine (10 mg/kg), BDNF expression levels and the *p*-CREB/CREB ratio in the GH and Flu groups were significantly higher than the CUMS group (*p* < 0.01, *p* < 0.01, respectively), suggesting that GH may regulate the cAMP-PKA-CREB-BDNF signal pathway to play an antidepressant role.

## Discussion

Depression is a mental disease characterized by constant low mood, loss of interest, anhedonia, unresponsiveness and sleeplessness (Wang et al., 2017). The causes of depression are complex, among which the increasing competitive pressure, unreasonable living habits and weak adaptability to the social environment are more crucial inducing factors (Wang et al., 2020). The CUMS is a mature animal model of depression that can simulate the chronic stress encountered by depressed patients in clinical observation (Yang et al., 2018). Currently, the widely used assays for depressive-like behaviors include the Sucrose preference test (SPT) and the Open field test (OFT). The SPT could mimic anhedonia-like behavior, a core symptom of depression in human (Sideromenos et al., 2020), and the OFT was used to determine general activity and exploratory behavior, signs of depression (Adelöf et al., 2018). Thus, the study established CUMS rat model and conducted a series of behavioral tests, including SPT and OFT, to study the effects of GH on antidepressant-like behaviors. The results of behavioral indexes of rats in each group indicated that GH has antidepressant effects, and the middle dose of GH showed powerfully inhibitory effects on rats with depressant-like behavior induced by CUMS.

Based on the characteristics of multi-channel and multi-target effects of Chinese herbal compound, the monoamine neurotransmitters such as DA, NE and 5-HT were regarded as the biochemical indexes to further study the antidepressant effects of GH (Gu et al., 2018). The monoamine hypothesis believes that the deficiency of major monoamine neurotransmitters (5-HT, DA, and NE) will lead to decrease of neurotransmission in the brain and the impairment of cognitive performance which may lead to depression (Kofler et al., 2019). Therefore, this study established a CUMS rat model for five weeks to observe the effects of GH intervention on the depressive-like behaviors and the changes of the monoamine neurotransmitters levels in the hypothalamus. The results of biochemical indexes of rats in each group indicated that GH has antidepressant effects, and middle dose of GH had stronger antidepressant effect. In the following mechanism verification experiment, the study mainly focused on the treatment group of GH at the middle dose (56 mg/kg).

Chromatographic techniques coupled with mass spectrometry has been an available method for rapid identification of components in Chinese medicine (Guo et al., 2018). UPLC/IT-TOF MS analysis was performed to identify the chemical components in GH. In this study, the 28 chemical components of GH were identified. Among the chemical components, only 20(*S*)-ginsenoside Rh2 was selected as active ingredient which satisfied the screening rules, OB ≥ 30% and DL ≥ 0.18. It is consistent with previous studies that ginsenoside Rh2, a main component in GH (Zhou et al., 2014), was a detectable compound in human plasma (Yue et al., 2019), and it can be filtered out by cell membrane chromatography (Ma et al., 2018).

To investigate the pharmacological mechanism of antidepressant effects of GH, 20(*S*)- ginsenoside Rh2, the active ingredient of GH, was used for network pharmacological analysis, and the top 10 significant signaling pathways were enriched by KEGG according to the *p* value. Based on literature search, the top 10 significant signaling pathways could involve in the occurrence of depression in varying degrees. For example, pathways in cancer and proteoglycans in cancer indirectly change the probability of depression by influencing HPA axis function (Young and Singh, 2018); Neuroactive ligand-receptor interaction, cocaine addiction and dopaminergic synapse could affect the occurrence of depression through regulating dopamine levels, emotion, learning and memory functions (Haile et al., 2007; Yang et al., 2017; Sun et al., 2018); cAMP signaling pathway, PI3K-Akt signaling pathway, estrogen signaling pathway, glutamatergic synapse and gap junction could intervene the occurrence of depression by mediating neural plasticity (Hennebelle et al., 2014; Crider and Pillai, 2017; Peng et al., 2018; Ren et al., 2018; Wu et al., 2018).

At present, the antidepressant mechanism of GH is unknown. Among the top 10 signaling pathways predicted by network pharmacological, the signaling pathway ranking at the top had strong correlation with the antidepressant effects of GH, which is the main signaling pathway of the antidepressant mechanism of GH. Therefore, the signaling pathway ranking at the top has more research value (Xiong et al., 2020). The first signaling pathway is neuroactive ligand-receptor interaction, which was related to all receptors and ligands associated with intracellular and extracellular signaling pathways in the plasma membrane (Pan et al., 2011). However, the neuroactive ligand-receptor interaction signaling pathway was related to the occurrence of various diseases, the mechanism of action on depression is not specific, and there are only two targets of receptor and ligand. Consequently, the first signaling pathway was not selected to further research. The second signaling pathway is cAMP signaling pathway, which was the most widely studied signaling pathway in the mechanism of antidepressant effects. In the cAMP signaling pathway, BDNF, the terminal downstream protein neurotrophic factor, could resist the damage of neurons, promote the repair and regeneration of neurons, and increased the secretion of monoamine neurotransmitters. Hence, Stress events compromise neuroplasticity via reduction of BDNF and lead to the occurrence of depression (Schmitt et al., 2016). According to the literature, neuroimaging and post-mortem studies have revealed impaired cAMP signaling in depressive patients, indicating cAMP signaling pathway was significantly associated with depression (Plattner et al., 2015). Thus, cAMP signaling pathway is selected to study the antidepressant mechanism of GH.

It is extremely clear that the mechanism of the cAMP signal pathway on depression. Under normal physiological conditions, monoamine neurotransmitters (such as 5-HT, DA, NE) interacts with specific G protein-coupled receptors (including 5-HT receptors, DA receptors, NE receptors) on the cell membrane to activate G protein (Cabrera-Vera et al., 2003; Fredriksson et al., 2003). The G-protein binds to Guanosine triphosphate (GTP) and subsequently GTP-G protein binds to the C2 domain and activates the adenylate cyclase (AC) enzyme. Activated AC catalyzes the biosynthesis of cAMP from adenosine triphosphate (ATP) (Frezza et al., 2018). The cAMP binds to the regulatory subunits of PKA (a tetramer consists of two regulatory subunits and two catalytic subunits), which resulted in the dissociation of catalytic subunit of PKA and enter to the cell nucleus (Wang et al., 2018). In the nucleus, the catalytic subunit of PKA binds to the Ser-133 site of CREB and phosphorylates CREB (Tu et al., 2019). Phosphorylated CREB (*p*-CREB) combines with the cAMP responsive elements (CRE) in the promoter region of BDNF, which can regulate BDNF transcription (Björkholm and Monteggia, 2016). Under the pathological conditions, continuous mental stress and stimulation would lead to the decrease in the content of monoamine neurotransmitters in the patient’s body and weaken the transduction of cAMP signaling pathway, which cause the decrease of BDNF expression (Zhang et al., 2012). Lower expression of BDNF is difficult to resist the injury of neurons under stress, which could lead to depression (van den Buuse et al., 2020).

**FIGURE 7:**
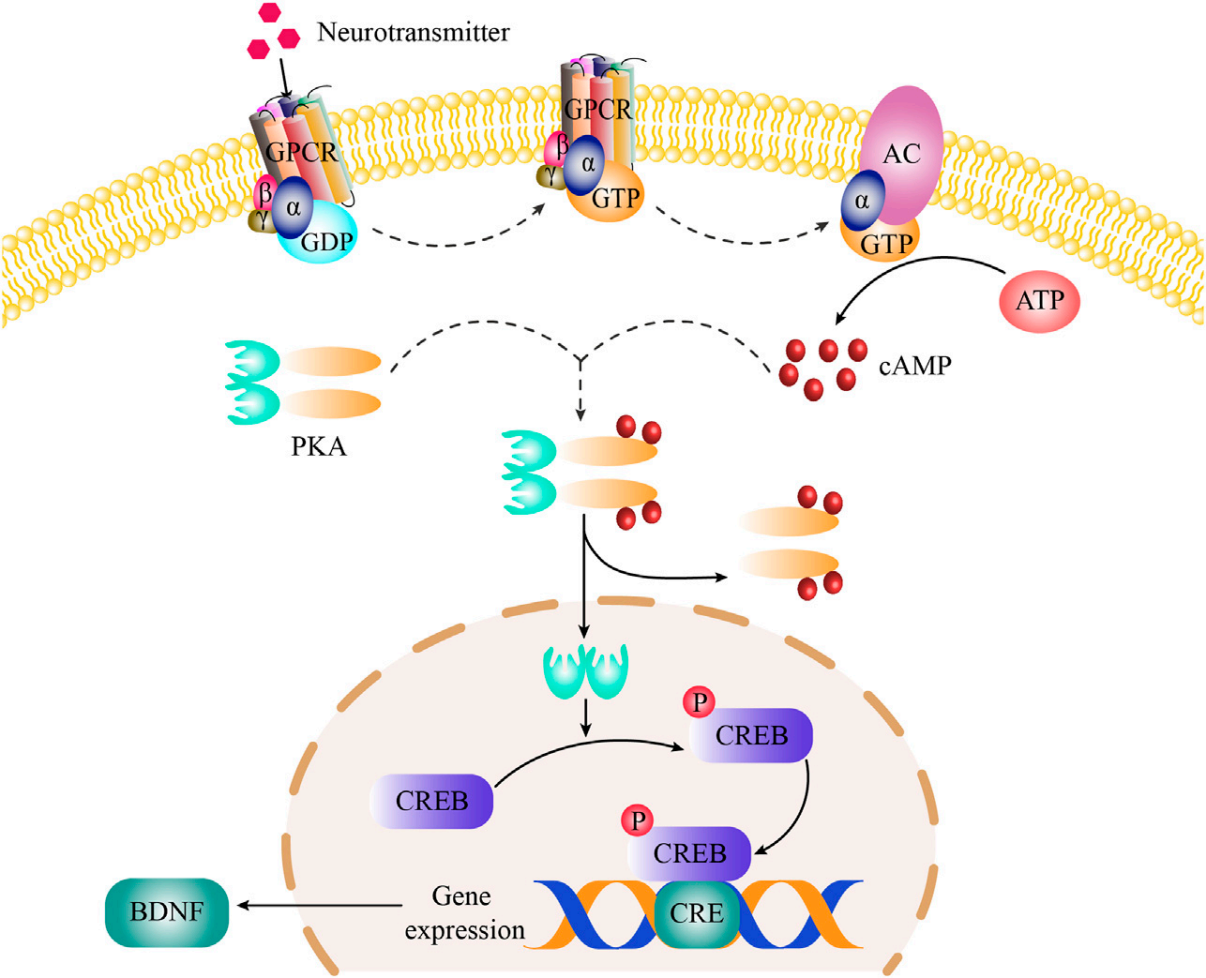
The cAMP-PKA-CREB-BDNF signaling pathway.

In cAMP signaling pathway, 11 target proteins, such as BDNF, GRIA1, CREB1, ADORA1 and so on, were enriched. The high-degree target proteins in the network may account for the essential therapeutic effects of GH on depression (Guo et al., 2019). The degree value of BDNF is the largest, indicating that BDNF is the most important in cAMP signaling pathway. BDNF is related to the survival, growth, and differentiation of neurons, and plays an important role in the signal transduction of depression (Wang et al., 2017). As the upstream protein of BDNF, *p*-CREB could regulate the expression of BDNF. At the same time, CREB has been confirmed to be related to the pathogenesis of depression and is one of the transcription factors with the most research on antidepressant effects (Wang et al., 2018). Therefore, Western blot was used to determine the expression level of BDNF and the ratio of *p*-CREB/CREB to study the antidepressant mechanism of GH. The results showed that GH significantly increased the expression level of BDNF and the ratio of *p*-CREB/CREB in the hippocampus of CUMS model rats. In order to further study the antidepressant mechanism of GH, the radioactive PKA assay kit was used to assay the activity of PKA which is the upstream protein of CREB. ELISA was used to detect the contents of cAMP which is the activator of PKA. As shown in our results, GH significantly upregulated the activity of PKA and the content of cAMP in hippocampus of CUMS model rats. Therefore, GH may play an antidepressant effects by regulating cAMP-PKA-CREB-BDNF signaling transduction. The cAMP-PKA-CREB-BDNF signaling pathway was presented in Figure 7.

Fluoxetine, a serotonin reuptake inhibitor (SSRI), is mainly used in the treatment of depression in clinic, which can improve the content of 5-HT. The 5-HT combined with its receptors, which resulted in that the cAMP signal pathway is activated and the levels of cAMP, PKA and the ratio of *p*-CREB/CREB were increased (Mato et al., 2010; Zhang et al., 2018). Then the expression level of BDNF is increased, which resisted the injury of neurons under stress, and exert antidepressant effects (Xie et al., 2019). During this process, BDNF increased the content of monoamine neurotransmitters by improving the activity of dopaminergic neurons and noradrenergic neurons, which is more conducive to play an antidepressant effects (Siuciak et al., 1996; Zhang et al., 2007), Therefore, fluoxetine was selected as a positive drug to explore whether GH exerts antidepressant effects by activating the cAMP signal pathway. The results indicated that GH could activate cAMP signaling pathway to play an antidepressant role, but whether it has the same antidepressant mechanism as fluoxetine through increasing 5-HT content and activating cAMP signaling pathway remains to be further studied.

## Conclusion

In the present study, an integrative pharmacology-based pattern, which adopted pharmacodynamics-network pharmacology-mechanism verification, was used to uncover the pharmacological mechanism of GH against depression. Firstly, it was found that GH at the middle dose (56 mg/kg) obviously alleviated depression-like behaviors induced by CUMS and showed powerful antidepressant effects. Then, we identified 28 main chemical components of GH by UPLC/IT-TOF MS. Furthermore, network pharmacology analysis predicted that cAMP signaling pathway may be the potential pharmacological mechanism regulated by GH acting on depression. Finally, the cAMP signaling pathway was verified as the mechanism of GH against depression through experimental validation of the target proteins (cAMP, PKA, *p*-CREB, and BDNF). Taken together, the current study suggested that GH could exert antidepressant effects by activating the cAMP-PKA-CREB-BDNF signaling pathway in hippocampus, which provided an effective method to uncover the pharmacological mechanism of traditional Chinese medicine.

## Data Availability Statement

The raw data supporting the conclusions of this article will be made available by the authors, without undue reservation, to any qualified researcher.

## Ethics Statement

The animal study was reviewed and approved by Tianjin University of Traditional Chinese Medicine Animal Care Committee.

## Author Contributions

LZ performed the experiments, drafted and modified the manuscript. RG, NC, YL, and XM analyzed the data and modified the manuscript. SP and WY prepared the materials of the paper. YZ, YL, and ZS conceived or designed the studies. WD, XX, and CL contributed to research design, experimental setup, results monitoring, and manuscript correction. All the authors read and approved the final manuscript.

## Funding

The research was supported by the National Natural Science Foundation of China (81973557), Natural Science Fund of Tianjin City (20JCZDJC00010), and the National Major Scientific and Technological Special Project of China (2018ZX09303-024, 2018ZX09737-019).

## Conflict of Interest

The authors declare that the research was conducted in the absence of any commercial or financial relationships that could be construed as a potential conflict of interest.

## Footnotes

1
http://lsp.nwu.edu.cn/tcmspsearch.php

2
http://www.swisstargetprediction.ch/

3
http://www.drugbank.ca/

4
http://db.idrblab.net/ttd/

5
http://www.omim.org/

6
https://string-db.org/

7
https://david-d.ncifcrf.gov/
